# Feasibility of smartphone app-based neuropsychological tasks for screening people with subclinical depression and anxiety: a preliminary validation study

**DOI:** 10.3389/fpsyt.2026.1773101

**Published:** 2026-03-16

**Authors:** Mingyu Jeon, Sanghun Lee, Yongjun Lee, Soo-Bin Uam, Yong Min Ahn, Min-Sup Shin

**Affiliations:** 1 Department of Psychiatry, Seoul National University Hospital, Seoul, Republic of Korea; 2 School of Psychology, Korea University, Seoul, Republic of Korea

**Keywords:** app-based screening tool, neuropsychological task, depression, anxiety, validity

## Abstract

**Introduction:**

Early identification and intervention for individuals at elevated risk for mental disorders is critical for improving quality of life and reducing social costs. Conventional self-report screening tools, however, are susceptible to social desirability and recall biases. Therefore, this study explored the feasibility and validity of smartphone app-based neuropsychological tasks designed to complement self-report measures and assist in the early screening of individuals with subclinical depression and anxiety.

**Methods:**

A subclinical depression/anxiety group (n = 55) and a control group (n = 57), aged 19–50 years (mean age = 36.14 ± 8.34), completed app-based neuropsychological tasks. Criterion-related validity was assessed using Pearson correlations between task scores and self-report scales measuring depression, anxiety, self-esteem, negative rumination, anxiety sensitivity, and distress intolerance. Discriminant validity was evaluated by conducting independent sample t-tests. Finally, discriminant analysis was performed using task variables that significantly differed between groups to evaluate classification accuracy.

**Results:**

Several index scores from the app-based tasks were significantly correlated with depression, anxiety, and related self-report measures. Mean differences between the subclinical and control groups were also significant. Discriminant analysis using auditory working memory, abandonment tendency, motivational deficit, and reasoning accuracy scores from the app-based tasks yielded a classification accuracy of 70.5% (leave-one-out cross-validation = 67.0%). When both neuropsychological task scores and depression- and anxiety-related self-report measures were included as independent variables, the classification accuracy increased to 91.1%.

**Discussion:**

The findings suggest that app-based neuropsychological tasks may serve as a promising adjunctive tool for early screening of individuals with subclinical depression and anxiety, addressing limitations associated with self-report measures.

## 1 Introduction

Depression and anxiety are among the most common and consequential mental disorders, exerting substantial impacts on the quality of life, occupational functioning, and social participation of affected individuals worldwide. The World Health Organization (WHO) reported that approximately 4% of the global population experiences depression, with an adult-point prevalence of 5.7% (6.9% in women; 4.6% in men) (1). These conditions also incur significant societal costs. An economic analysis estimated that every US$1 invested in treatment for depression and anxiety yields roughly US$4 in combined health and productivity benefits (2). In the United States, the annual socioeconomic burden of major depressive disorder (MDD) increased by 37.9%, from US$236.6 billion in 2010 to US$326.2 billion in 2018 (3). The coronavirus disease 2019 (COVID-19) pandemic further magnified this burden, with a large-scale analysis indicating an approximately 25% increase in the prevalence of depression and anxiety worldwide during the first year of the pandemic (4). Comorbidity between depressive and anxiety disorders is also among the highest in psychiatric diagnoses (5–7), associated with more complex illness courses, greater functional impairment and healthcare utilization, and poorer prognosis (8–10). Randomized controlled evidence suggests that strengthening primary care detection and referral programs can improve depressive symptom severity, as well as response and remission rates (11), underscoring the public health value of promptly identifying high-risk individuals and linking them to appropriate interventions.

In clinical settings, self-report instruments remain the most widely used tools for screening depression and anxiety. Standardized scales such as the Patient Health Questionnaire-9 (PHQ-9), Generalized Anxiety Disorder 7-item (GAD-7), and Beck Depression Inventory-II are easy to administer, score, and interpret; inexpensive; and available in multiple languages, which facilitates large-scale deployment (12–14). Nevertheless, self-report measures have well-recognized limitations. Social desirability and concerns about stigma can distort responses (15), and reference group effects can lead to different interpretations of the same scores across cultures and subpopulations (16–18). Moreover, retrospective reports at a single time point cannot fully capture intraday or day-to-day fluctuations and contextual effects (19). Discrepancies between ecological momentary assessment (EMA) and retrospective recall have been observed among patients with depression (20). Accordingly, the accuracy and interpretability of self-report scores are enhanced when they are triangulated with information from clinical interviews and observations, performance-based indices, and digital markers such as EMA data (21, 22).

Neuropsychological tasks offer a complementary approach by directly indexing cognitive and affective processing through performance metrics—for example, accuracy, errors, response times, and choice patterns—elicited in standardized stimulus–response contexts. As such, they are less susceptible to response strategies and social desirability and recall biases (15, 22). Previous studies have leveraged task-based measures to delineate the characteristics of depression and anxiety. For depression, meta-analytic evidence indicates broad impairments in executive functions (e.g., attentional shifting, inhibition, working memory) (23, 24), and effort-based decision-making and reward-learning paradigms suggest reduced motivation and diminished effort investment (25). Regarding anxiety, threat-related verbal and facial stimuli modulate attention and working memory, and decision-making contexts often reveal heightened sensitivity to punishment signals and avoidant tendencies (26). Such performance-based indicators can objectively capture the executive and motivational features of depression, as well as threat-related attention and avoidant decision-making in anxiety, and may provide convergent evidence to corroborate self-report findings (5, 24–28).

Smartphone app-based assessment can potentially mitigate the structural limitations of traditional, in-person testing. In recent years, tablet- and personal computer (PC)-adapted neuropsychological tools have been introduced; McIntyre et al. reported that a tablet/PC-based instrument could rapidly screen cognitive deficits in adults with MDD (29). For depression, mobile self-report screening has been the predominant approach (30, 31), but recent research has begun to explore digital phenotyping using smartphones and wearables for screening depression and anxiety (32).

However, empirical studies implementing neuropsychological tasks in smartphone app environments to screen for depression and anxiety remain limited. Usability research on digital neuropsychological assessments suggests advantages in speed, efficiency, and error reduction (33); yet, many mental health apps still focus primarily on self-monitoring and psychotherapy modules (often cognitive behavioral therapy), with comparatively few adopting performance-based tasks (34–36). Furthermore, existing task-based studies have often targeted single tasks or isolated cognitive domains, such as response time or working memory, rather than integrating the multidimensional neurocognitive features associated with depression and anxiety (37). These gaps highlight the need for additional validation of smartphone-based neuropsychological assessment tools in practical settings.

Synthesizing the theoretical background and prior findings, smartphone-based neuropsychological tasks appear promising for the early identification of individuals at elevated risk for depression and anxiety. App-based assessment can reduce the temporal, spatial, and economic constraints of in-person evaluations and may help reduce clinician time burden while improving workflow (33). As neuropsychological tasks deliver standardized stimuli, they can reduce the bias inherent in subjective self-reports, and automated administration and scoring can minimize human error and enhance data reliability (38). Therefore, app-based task batteries may complement existing screening and improve the overall quality of mental health services.

Accordingly, we developed a set of smartphone app-based neuropsychological tasks to facilitate early screening for subclinical depression and anxiety. In this study, ‘subclinical’ is operationally defined based on cut-off scores from self-reported scales (PHQ-9 and GAD-7) rather than formal diagnostic interviews, focusing on individuals who elevate on symptomatic measures but may not meet full clinical criteria. Drawing on established neuropsychological literature, the tasks were designed to comprehensively assess functions linked to depression and anxiety: working memory, risk-taking in decision-making, reasoning, negative cognitive bias, and motivational level. Through a multilayered validation process, we sought to explore the feasibility of using app-based neuropsychological tasks as an initial screening tool for subclinical depression and anxiety and identify task-derived indicators that could contribute to group classification.

## 2 Methods

### 2.1 Participants

Data were collected from 120 participants (60 subclinical; 60 controls) during the recruitment period from January 3 to July 16, 2025. Eight datasets were excluded owing to app-related errors; thus, 112 participants were included in the final analyses.

Regarding the inclusion criteria for study participation, individuals in the subclinical depression/anxiety group met the following criteria: (1) aged 19–50 years; (2) high school education or higher; (3) ability to independently complete questionnaires and use a smartphone; (4) capacity to understand the study’s purpose and procedures and provide voluntary consent; and (5) a score ≥10 on the PHQ-9 and/or GAD-7. This decision to aggregate depression and anxiety into a single ‘subclinical’ group was made not only to facilitate early screening for high-risk individuals but also to reflect the high rate of comorbidity between these conditions, ensuring the tool’s practical utility in real-world clinical or subclinical settings. The control group met the same criteria except that both the PHQ-9 and GAD-7 scores needed to be ≤4. The exclusion criteria were as follows: (1) inability to read the informed consent document; (2) history of epileptic seizures; (3) current brain injury or neurological disorder; (4) diagnosis of or current treatment for intellectual disability; (5) diagnosis of or current treatment for schizophrenia; and (6) any other clinically significant finding that, in the judgment of the principal investigator or designated staff, rendered the individual unsuitable for participation.

### 2.2 Procedure

Participants were recruited through online advertisements. Individuals who expressed interest completed an informed consent form and a brief pre-screening survey (PHQ-9 and GAD-7).

Among respondents who satisfied the criteria for either the subclinical or control group, the research team conducted a telephone interview to reconfirm the inclusion and exclusion criteria. Candidates who were deemed eligible completed smartphone app-based tasks in person in a one-on-one setting administered by researchers with master’s-level training in clinical psychology.

All participants used the same earphones (SAMSUNG GALAXY BUDS FE) and smartphone model (SAMSUNG GALAXY S23) provided by the research team. After receiving standardized instructions, participants entered their demographic information and completed the app-based neuropsychological tasks in the following fixed order: working memory task, risk-taking decision-making task, word memory task, and visual tracking task, followed by self-report questionnaires. The session lasted approximately 30 min. Performance data were automatically stored on the smartphone and subsequently exported, de-identified with a non-identifiable ID, and stored in a Google Sheet accessible only to the research team.

This study represents the second-year work of the project “Development of a Multi-Modal Mental Health High-Risk People Screening System Using Neuropsychological Tests and Biosignals,” funded by the Korean Ministry of Science and ICT. The protocol was approved by the Institutional Review Board of Seoul National University Hospital (IRB No. H-2411-052-1585) prior to study commencement.

### 2.3 Measures

#### 2.3.1 Neuropsychological tasks

The descriptions of each task are provided as follows. Illustrative images and implementation details are presented in **Table 1**, and the theoretical background for each indicator is summarized in **Table 2**.

**Table 1:** Measured constructs and pictures of neuropsychological tasks.

Tasks	Measured constructs	Pictures
Working Memory Task	Visual/Auditory Working Memory, Abandonment Tendency, Response Time	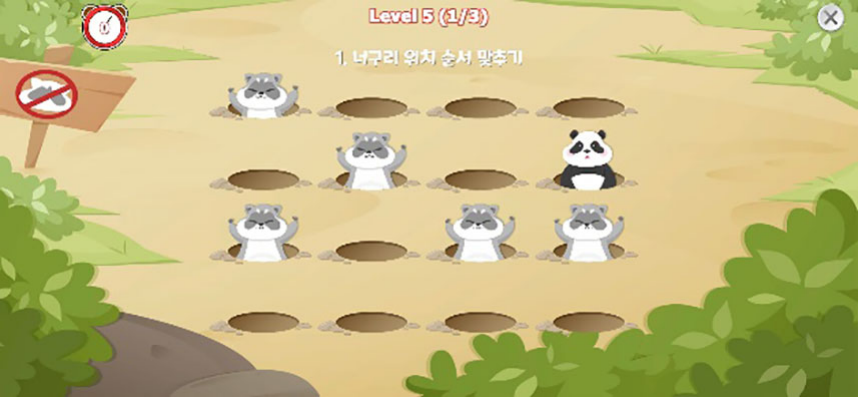 Memory of target (raccoon) appearance sequence 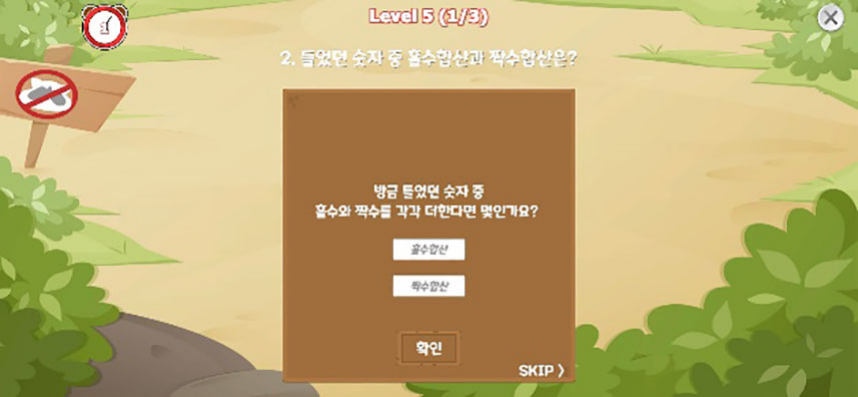 Calculation of odd/even count or sum
Risk-Taking Decision-Making Task	Risk-Taking Tendency, Decision Variability, Reasoning Accuracy, Motivational Deficit, Response Time	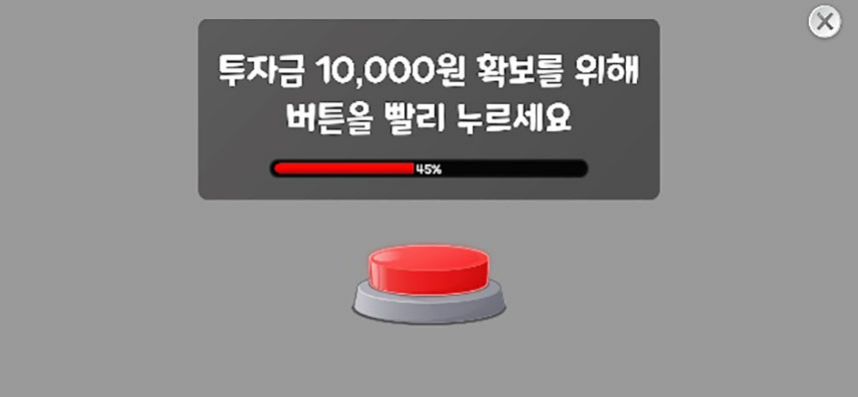 Rapid clicking for initial fund acquisition 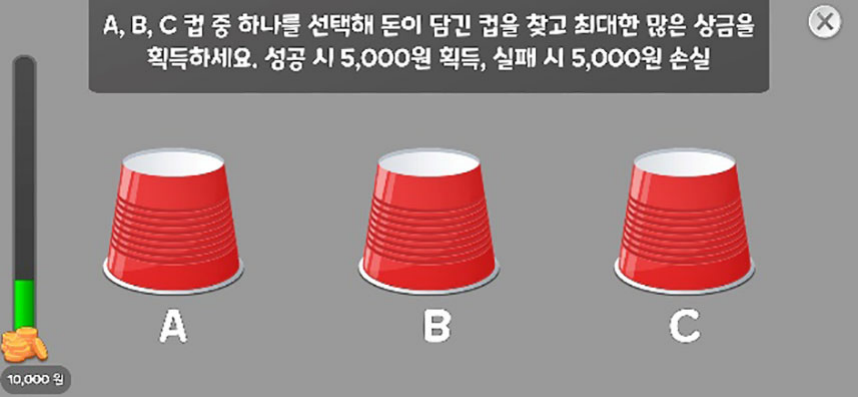 Cup selection among three options
Word Memory Task	Short-Term Memory, Negative Cognitive Bias, Response Time	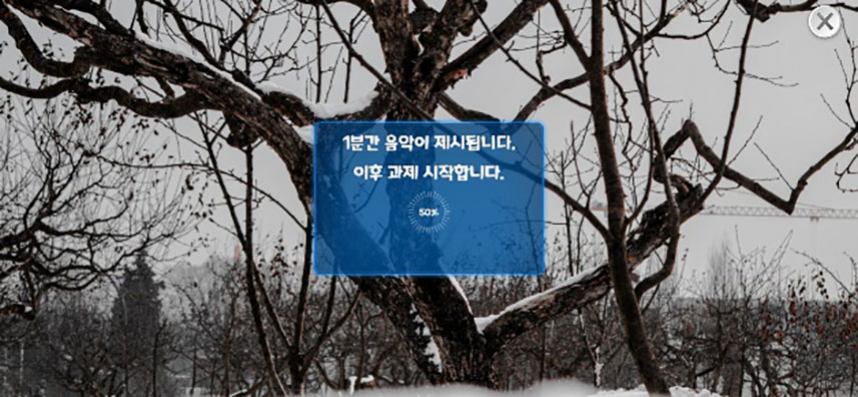 Negative mood induction 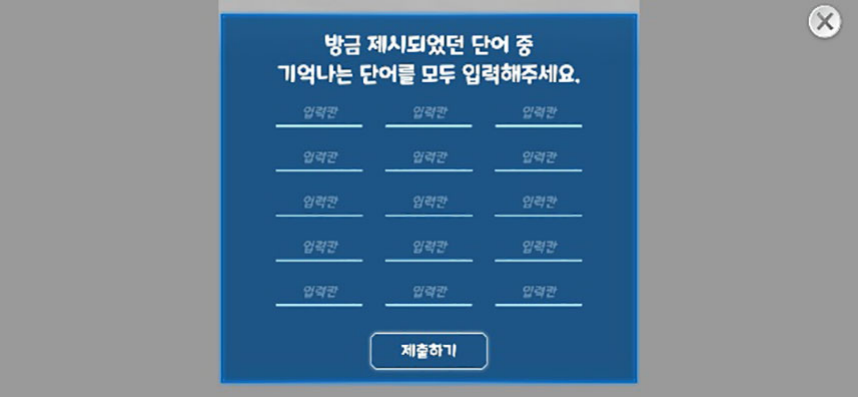 Word retention and free recall
Visual Tracking Task	Motor Effort, Visual Tracking Ability, Response Time	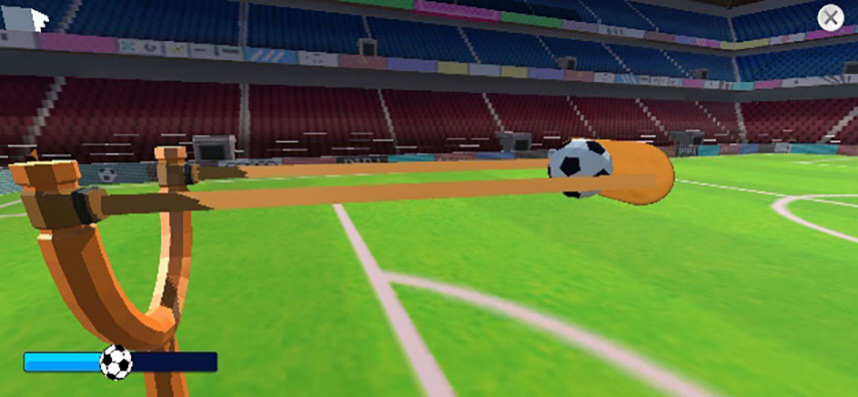 Slingshot loading and ball launching 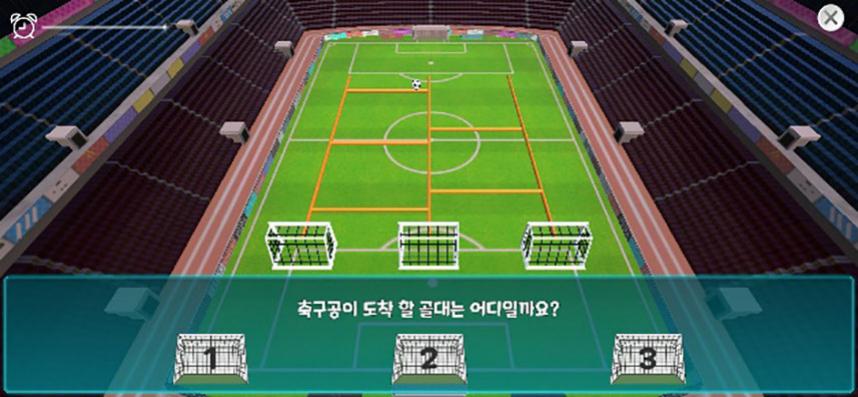 Prediction and selection of goal entry

**Table 2:** Overview of tasks, constructs, and theoretical foundations used in the app.

Tasks in the app	Construct	Similar tasks	Relevant theories and models	Corresponding research citations
Working Memory Task	Visual/Auditory Working Memory	- Corsi-block-tapping task - WAIS Digit Span & Arithmetic	- Cognitive Interference Theory [Attentional Control Theory (39)], Processing Efficiency Theory (40)] - Hedonic Defector Theory (41)	- (42) - (43) - (44)
	Abandonment Tendency	- Demand Selection Task (DST) - Non-response, pass, or skip in most tasks	- Cognitive Interference Theory (*same as the above reference*) - Mood-Behavior Model (45)	- (46) - (47)
Risk Taking Task	Reasoning Accuracy	- Object Alternation Test (OAT) - Iowa Gambling Test (IGT) - Wisconsin Card Sorting Test (WCST)	- Cognitive Interference Theory (*same as the above reference*) - Attention Distractibility (48) - Emotional Distraction (49)	- (50) - (51) - (52)
	Risk Taking Tendency	- Object Alternation Test (OAT) - Iowa Gambling Task (IGT) - Balloon Analogue Risk Task (BART)	- Reinforcement Sensitivity Theory (53)	- (54) - (55) - (56)
	Decision Variability	- Object Alternation Test (OAT) - Probabilistic Selection Task (PST) - Iowa Gambling Task (IGT)	- Reinforcement Sensitivity Theory (*same as the above reference*)	- (57) - (58)
	Motivational Deficit	- Effort Expenditure for Rewards Task	- Cognitive Interference Theory (*same as the above reference*) - Mood-Behavior Model (*same as the above reference*)	- (46) - (47)
Word Memory Task	Short Term Memory	- Rey Auditory Verbal Learning Test (RAVLT)	- Cognitive Interference Theory (*same as the above reference*) - Hedonic Defector Theory (*same as the above reference*)	- (59) - (42)
	Negative Cognitive Bias	- Tasks Employing Mood Induction	- Beck’s Cognitive Model (60) - Associative Network Theory (61) and Mood Congruent Memory (62)	- (63) - (64)
Visual Tracking Task	Motor Effort	- Grip Strength Effort Task (GSET)	- Cognitive Interference Theory (*same as the above reference*) - Mood-Behavior Model (*same as the above reference*)	- (46) - (47)
	Visual Tracking Ability	- Maze Task - Trail Making Test (TMT) Type A	- Cognitive Interference Theory (*same as the above reference*) - Psychomotor Retardation (65)	- (66)
Response time in all tasks	Response Time	- Response time in existing tasks	- Cognitive Interference Theory (*same as the above reference*)	- (67) - (68)

##### 2.3.1.1 Working memory task

This multitasking paradigm required participants to (a) encode and reproduce the sequence in which a target stimulus (a raccoon) appeared within an array of holes that increased in number across stages from 4 (2×2) to 16 (4×4), while simultaneously (b) attending to auditory number stimuli (1 or 2) and subsequently computing either the count of odd or even numbers or the sum of odd and even numbers. With advancing stages, the number of visual and auditory stimuli increased and distractors (a panda, non-target) were presented, thereby raising task difficulty. Participants could press a give-up button to proceed to the next stage if desired.

The working memory task yielded the following indicators: visual working memory, auditory working memory, and abandonment tendency. Visual working memory was captured by the number of correct reproductions across 14 visual-sequence trials. Auditory working memory was assessed by the number of correct responses across 22 auditory computation trials involving odd/even counts or sums. Higher scores indicate better working memory for visual and auditory stimuli. Abandonment tendency was computed as the proportion of trials in which performance was voluntarily discontinued; higher scores indicate more frequent quitting within given trials. Additionally, we calculated the average response time per item for the visual and auditory working memory questions that were attempted without quitting.

##### 2.3.1.2 Risk-taking decision-making task

In this task, participants first rapidly clicked a button to obtain initial funds, then chose between a high-risk/high-reward option (± 5,000 KRW) and a low-risk/low-reward option (± 1,000 KRW). Next, they selected one of three cups to either win or lose money, with opportunities to switch between the two options during the task. Across stages, the pattern of the correct location changed; no explicit rule cues were given, and participants were expected to infer the rule to optimize their choices.

The task produced the following indicators: reasoning accuracy, risk-taking tendency, decision variability, and motivational deficit. Reasoning accuracy was captured by the proportion of trials (out of 20) in which a reward was obtained; higher values indicate better inference of the reward–punishment rule. Risk-taking tendency was assessed by the proportion of choices favoring the high-risk/high-reward option across 20 trials; higher values indicate greater risk-taking. Decision variability was computed as the proportion of opportunities (out of 19) in which participants switched between the high-risk/high-reward and low-risk/low-reward options; higher values indicate more frequent switching and greater decision variability. Motivational deficit was indexed by the time (in seconds) required to press the button to obtain initial funds; higher values indicate slower motor speed for obtaining initial funds and greater motivational deficit. We also computed the average response time per trial.

##### 2.3.1.3 Word memory task

In this task, participants first underwent negative mood induction: With sad music and background images, they were instructed to recall a past sad or difficult event and speak about it for 1 min. Thereafter, they encoded a list of words and performed free recall to report as many words as possible. Next, positive mood induction was conducted to counteract negative affect by having participants recall a happy, pleasant event and speak about it for 1 min; the same encode-and-recall procedure was repeated. In both the negative and positive mood-induction conditions, word presentation and recall were each repeated three times. For each condition, 15 words were used (five neutral, five negative, and five positive).

This task yielded the indices of short-term memory and negative cognitive bias. Short-term memory was computed as the proportion of correctly entered words across 90 total entry opportunities; higher values indicate better verbal short-term memory. Negative cognitive bias was defined as the proportion of negative words among all successfully recalled emotional (negative or positive) words; higher values indicate a stronger tendency to recall negative words, consistent with negative cognitive bias. We also calculated the average writing time per word.

##### 2.3.1.4 Visual tracking task

In this task, participants pulled back the string of a slingshot to launch a ball and rapidly predicted the goal that the ball would enter while descending along multiple ladders. As the stages advanced, both the ball speed and number of ladders increased, requiring greater attentional focus and faster visual tracking.

This task produced the indices of motor effort and visual tracking ability. Motor effort ranged from 1 to 100 and was based on the distance the slingshot was pulled for launching; higher values indicate a longer pull. Visual tracking ability was defined as the proportion of correct predictions (out of 13 trials) of the goal into which the ball would fall; higher values indicate better visual tracking. We also calculated the average time per action for the shooting and goal-selection phases.

#### 2.3.2 Self-report measures

PHQ-9 and GAD-7 were used as prescreening tools to classify participants into subclinical and control groups. We administered the following digitized self-report scales to evaluate criterion-related validity by testing whether the app-based neuropsychological indicators aligned with theoretically related self-reported constructs (depression, anxiety, self-esteem, ruminative responses, anxiety sensitivity, and distress intolerance).

##### 2.3.2.1 PHQ-9

On the PHQ-9 (13), the Korean adaptation of which was used in this study (69), respondents rate the frequency of nine depressive symptoms that correspond to the Diagnostic and Statistical Manual of Mental Disorders (DSM) diagnostic criteria for MDD on a scale of 0−3, yielding a total score of 0–27. Higher scores indicate greater depressive severity; the severity categories are 0–4, none; 5–9, mild; 10–14, moderate; 15–19, moderately severe; and 20–27, severe. In the present study, Cronbach’s α was 0.92 at screening and 0.93 during in-app administration.

##### 2.3.2.2 Patient-reported outcomes measurement information system level 2–depression–short form

The PROMIS Depression-SF (70), which has been translated into Korean (69), comprises eight items rated on a 1–5 frequency Likert scale. Total scores range from 8 to 40; higher scores indicate greater depressive severity. Severity may also be interpreted using T-scores corresponding to the raw scores. Cronbach’s α in this study was 0.97.

##### 2.3.2.3 Rosenberg self-esteem scale

We used the RSES (71) translated into Korean (72). The scale contains 10 items rated on a 1–4 Likert scale, with total scores ranging from 10 to 40. Items 2, 5, 6, 8, and 9 are reverse-scored. Higher scores reflect greater self-esteem. In a Korean adult sample, Cronbach’s α was 0.90; in the present study, it was 0.94.

##### 2.3.2.4 Ruminative response scale

The RRS comprises 22 items representing the “ruminative response style” subset of the Response Styles Questionnaire (73); the Korean version has been validated (74). Items are rated on a 1–4 Likert scale, yielding total scores ranging from 22 to 88. Higher scores indicate a stronger ruminative response style. In a Korean college sample, Cronbach’s α was 0.89 (74); in this study, it was 0.96.

##### 2.3.2.5 GAD-7

The GAD-7 (14), which has been translated into Korean (75), comprises seven items rated on a 0–3 frequency Likert scale for symptoms during the past two weeks, yielding a total score of 0–21. Higher scores indicate greater anxiety severity; the original developers proposed the following categories: 0–4, minimal; 5–9, mild; 10–14, moderate; and 15–21, severe. Internal consistency (Cronbach’s α) was 0.92 in the original version and 0.92 in a Korean adult sample. In this study, Cronbach’s α was 0.93 at screening and 0.95 at app administration.

##### 2.3.2.6 Patient-reported outcomes measurement information system level 2–anxiety–short form

The PROMIS Anxiety-SF (70), also translated into Korean (69), comprises seven items rated on a 1–5 frequency Likert scale. Total scores range from 7 to 35; higher scores indicate greater anxiety severity. Severity may also be interpreted using T-scores corresponding to the raw scores. Cronbach’s α in this study was 0.95.

##### 2.3.2.7 Anxiety sensitivity index-3

The ASI-3 (76) assesses the fear of anxiety-related sensations using 18 items rated on a 0–4 intensity Likert scale, with total scores ranging from 0 to 72; higher scores indicate greater anxiety sensitivity. Across diverse samples, subscale Cronbach’s α values in the original version ranged from 0.73 to 0.91. The Korean version has been validated (77), with Cronbach’s α = 0.87. In this study, Cronbach’s α was 0.94.

##### 2.3.2.8 Distress intolerance index

The DII (78), translated and validated in Korean (79), includes 10 items rated on a 0–4 Likert scale (total range: 0–40), with higher scores indicating lower distress tolerance. The original version reported Cronbach’s α values of 0.91–0.92; in a Korean college sample, Cronbach’s α was 0.87. In this study, it was 0.91.

### 2.4 Data analysis

#### 2.4.1 Validity

First, to examine the criterion-related validity of the app-based neuropsychological tasks, we conducted Pearson’s correlation analyses between task-derived indicators and self-report scale scores. The correlation set included the PHQ-9, PROMIS Depression-SF, RSES, RRS, GAD-7, PROMIS Anxiety-SF, ASI-3, and DII. We tested whether the task performance indices showed theoretically consistent associations with established clinical constructs (depression, anxiety, rumination, self-esteem, anxiety sensitivity, and distress intolerance).

Second, to evaluate the discriminant validity of the app-based neuropsychological assessment, we compared the task indicators between the control and subclinical depression/anxiety groups. Group differences were analyzed using independent sample t-tests to determine whether the two groups exhibited significant performance differences.

Finally, we performed discriminant analyses to estimate the accuracy of the developed app-based neuropsychological tasks in classifying the subclinical versus control status. As predictors, we first entered task variables that showed significant group differences in independent sample t-tests and quantified classification accuracy based on neuropsychological indicators alone. We then entered both task variables and self-report scales related to depression and anxiety (excluding the PHQ-9 and GAD-7) as predictors to estimate classification accuracy when combining task-based and self-reported indicators. To clarify the relative importance of each predictor in group separation, standardized canonical discriminant function coefficients were examined for both models. Statistical analyses were performed using IBM SPSS Statistics (version 22).

#### 2.4.2 Reliability

We assessed the internal stability of the app-based measures to ensure their reliability. Internal consistency (Cronbach’s α) was calculated for indicators with multi-trial structures, and Spearman-Brown corrected split-half reliability ( $r_{sb}$) was employed for tasks involving increasing difficulty or specific mood-induction phases. Due to the novel and performance-based nature of these digital tasks, these analyses were conducted as exploratory assessments of measurement stability.

## 3 Results

### 3.1 Study population

Demographic characteristics and screening-time depression and anxiety scores are presented in **Table 3**. Of the 120 enrolled participants, 8 were excluded due to app-related errors, and the final analytic sample comprised 112 individuals (subclinical group: n = 55; control group: n = 57). Group-comparison tests indicated no significant differences in gender distribution (χ² = 1.43, *p* = 0.23) or age (t = 0.98, *p* = 0.33). By contrast, both the PHQ-9 (t = 22.33, *p<* 0.001) and GAD-7 (t = 14.31, *p<* 0.001) scores differed significantly between groups, with the subclinical group showing higher depressive and anxiety levels.

**Table 3:** Sample characteristics and results of t-tests for each neuropsychological assessment variable.

Variable	Unit	Subclinical group (*n* = 55)		Control group (*n* = 57)		t/*χ²*	*p*
		*Mean*	*SD*	*Mean*	*SD*		
Sample characteristics							
Gender (n of Women)	n	36	–	31	–	1.43	0.23
Age	years	36.93	7.61	35.39	9.00	0.98	0.33
Self-report measures							
PHQ-9	score	14.22	3.92	1.95	1.33	22.33***	< 0.001
GAD-7	score	10.04	4.65	0.96	1.13	14.31***	< 0.001
Working memory task							
Visual Working Memory	n	8.64	1.96	8.89	2.01	-0.69	0.49
Auditory Working Memory	n	14.69	3.94	16.14	3.61	-2.03*	0.04
Abandonment Tendency	%	0.05	0.08	0.03	0.04	2.13*	0.04
Response Time	s/trial	7.39	1.60	6.97	1.86	1.28	0.20
Risk-taking decision-making task							
Risk-Taking Tendency	%	0.49	0.35	0.48	0.37	0.23	0.82
Decision Variability	%	0.17	0.19	0.13	0.13	1.41	0.16
Reasoning Accuracy	%	0.44	0.10	0.39	0.13	2.21*	0.03
Motivational Deficit	s	5.99	1.26	5.31	1.17	2.97**	< 0.01
Response Time	s/trial	6.32	1.05	6.48	1.15	-0.80	0.43
Word memory task							
Short-Term Memory	%	0.56	0.14	0.59	0.13	-1.15	0.25
Negative Cognitive Bias	%	0.50	0.07	0.48	0.06	1.57	0.12
Response Time	s/word	6.70	1.95	6.75	1.79	-0.15	0.88
Visual tracking task							
Motor Effort	score	89.00	11.39	86.60	12.68	1.05	0.30
Visual Tracking Ability	%	0.86	0.17	0.87	0.16	-0.02	0.98
Response Time	s/trial	3.04	0.54	2.92	0.54	1.17	0.24

*p<*.05 (*), *p<*.01 (**), *p<*.001 (***). PHQ-9, Patient Health Questionnaire-9; GAD-7, Generalized Anxiety Disorder-7. Bold values indicate statistical significance.

### 3.2 Reliability of task variables

To evaluate the internal stability and reproducibility of the app-based measures, reliability coefficients were calculated for all multi-trial indicators. As presented in **Supplementary Table 2**, most task variables demonstrated acceptable to excellent internal consistency, with coefficients ranging from 0.58 to 0.95.

High stability was particularly observed in the Motor Effort (α = 0.95) and Short-Term Memory (α = 0.87) tasks. While some indices, such as Reasoning Accuracy (α = 0.18) and Negative Cognitive Bias ( $r_{sb}$= 0.35), showed lower exploratory reliability, these values reflected the inherent variability of trial-and-error learning processes and the mathematical properties of proportion-based emotional metrics.

Notably, the components of the Word Memory Task—including Neutral, Positive, and Negative word recall—showed robust stability (α ranging from 0.68 to 0.77), confirming that the foundational data for the negative bias measures remained stable. For Reasoning Accuracy, the reliability increased from the first half (α = -0.19) to the second half (α = 0.22), indicating stabilized performance following the initial rule-inference phase.

### 3.3 Discriminant validity

Levene’s tests indicated that the assumption of homogeneity of variances was violated for some scales and indicators. However, the statistical significance of group differences did not change when accounting for heteroskedasticity. Accordingly, independent sample t-tests were conducted for all self-report scales and neuropsychological task indicators to examine performance differences between groups. The group means, standard deviations, t-statistics, and p-values are summarized in **Table 3**.

In the working memory task, the control group outperformed the subclinical group on auditory working memory (t = −2.03, *p* = 0.04), indicating higher auditory working memory accuracy in controls. Regarding abandonment tendency, the subclinical group showed a greater propensity to discontinue trials than the control group (t = 2.13, *p* = 0.04).

In the risk-taking decision-making task, the subclinical group achieved higher reasoning accuracy than the control group (t = 2.21, *p* = 0.03), indicating better identification of the reward-containing cup. For motivational deficit, the subclinical group pressed the button to obtain initial funds more slowly than the control group (t = 2.97, *p<* 0.01).

In the word memory task, although not statistically significant, the subclinical group showed a trend toward greater negative cognitive bias. Other indicators did not differ significantly between groups. No indicators showed significant group differences in the visual tracking task.

### 3.4 Criterion-related validity

**Figure 1:**
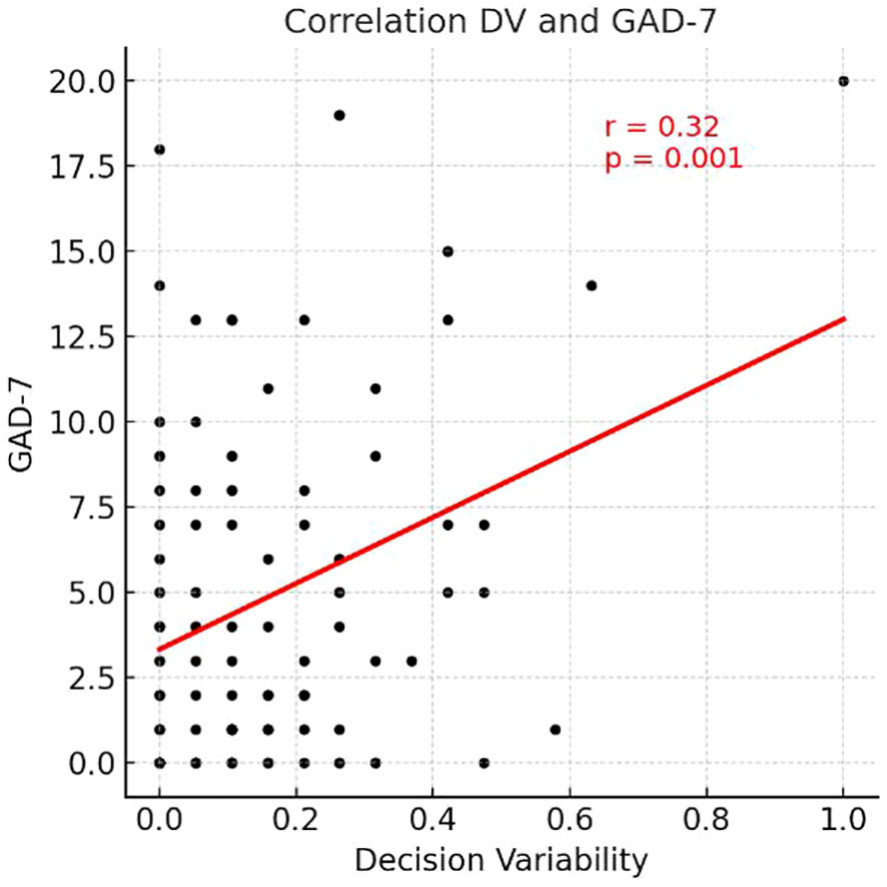
Scatterplot illustrating the correlation between Decision Variability and GAD-7 scores.

**Figure 2:**
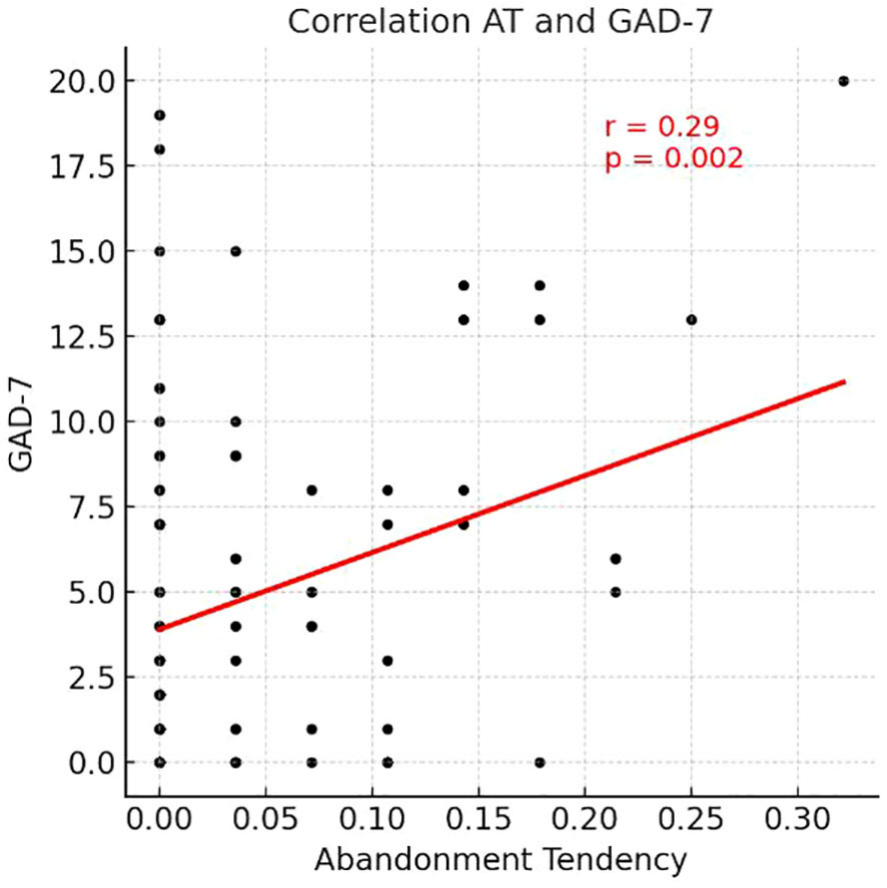
Scatterplot illustrating the correlation between Abandonment Tendency and GAD-7 scores.

**Figure 3:**
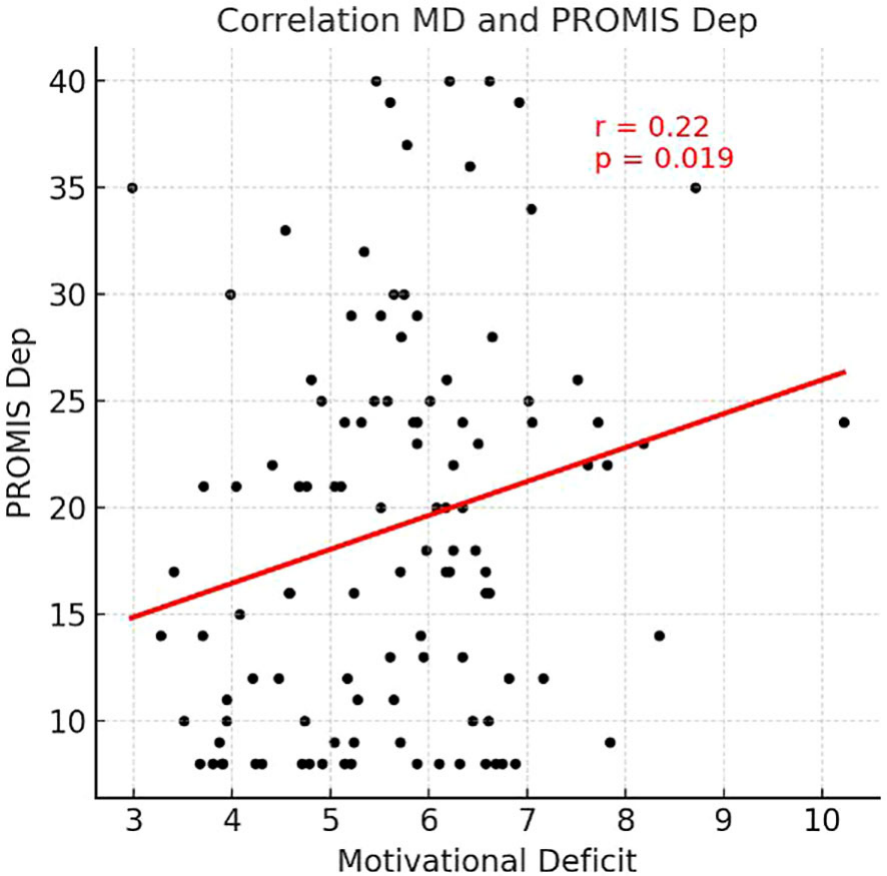
Scatterplot illustrating the correlation between Motivational Deficit and PROMIS Depression-SF scores.

Pearson’s correlations were computed to evaluate the criterion-related validity of the app-based neuropsychological indicators with self-report scales. Correlation coefficients for significant associations are presented in **Table 4**, and scatterplots for task indicators showing clear associations with the depression and anxiety scales are shown in **Figures 1**–**3**. The full correlation matrix between all task indicators and self-report measures is provided in **Supplementary Table 1**.

**Table 4:** Correlations among the study variables.

Item	PHQ-9	PROMIS Dep	RSES	RRS	GAD-7	PROMIS Anx	ASI-3	DII
1. A-WM	-.17	-.14	.20^*^	-.11	-.12	-.15	-.04	-.08
2. AT	.25^**^	.18	-.21^*^	.17	.29^**^	.25^**^	.21^*^	.26^**^
3. RA	.19^*^	.17	-.13	.18	.14	.14	.16	.13
4. DV	.22^*^	.17	-.19^*^	.14	.32^**^	.23^*^	.15	.10
5. MD	.20^*^	.22^*^	-.14	.06	.10	.21^*^	-.01	.03
6. R-RT	-.16	-.15	.13	-.18	-.17	-.12	-.19^*^	-.18
7. WD-RT	-.10	-.03	.10	-.19^*^	-.21^*^	-.10	-.21^*^	-.16

*N* = 112. *p* <.05 (^*^), *p* <.01 (^*^*). A-WM, Auditory Working Memory; AT, Abandonment Tendency; RA, Reasoning Accuracy; DV, Decision Variability; MD, Motivational Deficit; R-RT, Risk-Taking Decision-Making Task Response Time; WD-RT, Word Memory Task Response Time. PHQ-9, Patient Health Questionnaire-9; PROMIS Dep, PROMIS Depression Short Form; RSES, Rosenberg Self-Esteem Scale; RRS, Ruminative Response Scale; GAD-7, Generalized Anxiety Disorder-7; PROMIS Anx, PROMIS Anxiety Short Form; ASI-3, Anxiety Sensitivity Index-3; DII, Distress Intolerance Index. Bold values indicate statistical significance.

In the working memory task, auditory working memory accuracy was positively correlated with the RSES (r = 0.20, *p<* 0.05). Abandonment tendency was positively correlated with the PHQ-9 (r = 0.25, *p<* 0.01), GAD-7 (r = 0.29, *p<* 0.01), PROMIS Anxiety-SF (r = 0.25, *p<* 0.01), ASI-3 (r = 0.21, *p<* 0.05), and DII (r = 0.26, *p<* 0.01), and negatively correlated with the RSES (r = −0.21, *p<* 0.05).

In the risk-taking decision-making task, reasoning accuracy was positively correlated with the PHQ-9 (r = 0.19, *p<* 0.05). Decision variability was positively correlated with the PHQ-9 (r = 0.22, *p<* 0.05), GAD-7 (r = 0.32, *p<* 0.01), and PROMIS Anxiety-SF (r = 0.23, *p<* 0.05), and negatively correlated with the RSES (r = −0.19, *p<* 0.05). Response time for the risk-taking task was negatively correlated with the ASI-3 (r = −0.19, *p<* 0.05). Motivational deficit showed positive correlations with the PHQ-9 (r = 0.20, *p<* 0.05), PROMIS Depression-SF (r = 0.22, *p<* 0.05), and PROMIS Anxiety-SF (r = 0.21, *p<* 0.05).

In the word memory task, response time was negatively correlated with the RRS (r = −0.19, *p<* 0.05), GAD-7 (r = −0.21, *p<* 0.05), and ASI-3 (r = −0.21, *p<* 0.05).

Contrary to expectations, no significant correlations with the self-report scales were observed for indicators from the visual tracking task.

### 3.5 Classification accuracy

#### 3.5.1 Discriminant ability of neuropsychological task variables

We performed discriminant analysis to evaluate the classification accuracy between the subclinical and control groups using neuropsychological task indicators. As predictors, using the enter method, we entered four task variables that showed significant group differences (auditory working memory, abandonment tendency, reasoning accuracy, motivational deficit). Prior probabilities were set to be equal. The overall classification accuracy was 70.5%, with a sensitivity of 70.9% and a specificity of 70.2% (Wilks’ λ = 0.844, *p* = 0.001) (**Table 5**). To clarify which variables contributed most strongly to group separation, we examined the standardized canonical discriminant function coefficients. Motivational deficit showed the highest contribution (0.70), followed by reasoning accuracy (0.47), abandonment tendency (0.34), and auditory working memory (-0.33), providing insight into the relative importance of these digital markers for screening. Under the same settings, leave-one-out cross-validation yielded an accuracy of 67.0%, sensitivity of 65.5%, and specificity of 68.4%.

**Table 5:** Classification results based on neuropsychological task indicators.

Validation Type	Statistic	Group	Predicted group membership		Total
			Subclinical	Control	
Original	Count	Subclinical	39	16	55
		Control	17	40	57
	%	Subclinical	70.9	29.1	100.0
		Control	29.8	70.2	100.0
Cross-Validation	Count	Subclinical	36	19	55
		Control	18	39	57
	%	Subclinical	65.5	34.5	100.0
		Control	31.6	68.4	100.0

In total, 70.5% of the original grouped cases were correctly classified. Overall, 67.0% of the cross-validated grouped cases were correctly classified.

#### 3.5.2 Discriminant ability of neuropsychological task indicators plus self-report scales

To estimate classification accuracy when combining neuropsychological task indicators with self-report scales, we conducted discriminant analysis including, via the enter method, the four task variables that differed significantly between groups together with six self-report measures (PROMIS Depression-SF, RSES, RRS, PROMIS Anxiety-SF, ASI-3, DII). Prior probabilities were set to be equal. The overall classification accuracy was 91.1%, exceeding that obtained with tasks alone; sensitivity was 89.1% and specificity was 93.0% (Wilks’ λ = 0.377, *p<* 0.001) (**Table 6**). To identify the primary contributors to group separation, we examined the standardized canonical discriminant function coefficients. The most significant contributors were PROMIS Depression (0.66), Distress Intolerance (0.29), and Motivational Deficit (0.27). Other task-based and self-report indicators also contributed to the model, albeit with lower relative weights (see **Table 7** for the full list of coefficients). Under the same settings, leave-one-out cross-validation produced an accuracy of 88.4%, sensitivity of 87.3%, and specificity of 89.5%.

**Table 6:** Classification results based on neuropsychological task indicators and self-reports.

Validation Type	Statistic	Group	Predicted group membership		Total
			Subclinical	Control	
Original	Count	Subclinical	49	6	55
		Control	4	53	57
	%	Subclinical	89.1	10.9	100.0
		Control	7.0	93.0	100.0
Cross-Validation	Count	Subclinical	48	7	55
		Control	6	51	57
	%	Subclinical	87.3	12.7	100.0
		Control	10.5	89.5	100.0

In total, 91.1% of the original grouped cases were correctly classified. Overall, 88.4% of the cross-validated grouped cases were correctly classified.

**Table 7:** Standardized canonical discriminant function coefficients for the combined model.

Predictors	Standardized canonical discriminant function coefficients
Self-report measures	
PROMIS Dep	0.66
DII	0.29
PROMIS Anx	0.17
ASI-3	0.17
RRS	-0.16
RSES	-0.00
Task indicators	
Motivational Deficit	0.27
Auditory Working Memory	-0.19
Reasoning Accuracy	0.17
Abandonment Tendency	-0.04

PROMIS Dep, PROMIS Depression Short Form; RSES, Rosenberg Self-Esteem Scale; RRS, Ruminative Response Scale; PROMIS Anx, PROMIS Anxiety Short Form; ASI-3, Anxiety Sensitivity Index-3; DII, Distress Intolerance Index.

## 4 Discussion

The app-based tasks generally demonstrated acceptable to excellent internal stability, with reliability coefficients for most indicators ranging from 0.58 to 0.95 (see **Supplementary Table 2** for details). While lower exploratory reliability was observed in Reasoning Accuracy (0.18) and Negative Cognitive Bias (0.35), these were attributed to inherent learning dynamics and the mathematical properties of proportion-based metrics, respectively. Correlation analyses showed significant associations between three of the four tasks (all except the visual tracking task) and self-report measures indexing depression and anxiety symptoms or emotional vulnerability (e.g., self-esteem, anxiety sensitivity), suggesting preliminary criterion-related validity. Additionally, group comparisons indicated that relative to the control group, the subclinical group performed worse on the auditory working memory task, showed a higher tendency to abandon trials on the working memory task, and exhibited relatively lower motivational levels—patterns consistent with preliminary discriminant validity. Contrary to our expectations, however, the subclinical group demonstrated better reasoning accuracy than the control group on the risk-taking decision-making task. In discriminant analyses using neuropsychological tasks to classify the subclinical versus control status, the discriminant function was statistically significant, and the overall classification accuracy was 70.5%, representing a 20.5% improvement over chance (50%). Notably, Motivational Deficit (0.70) and Reasoning Accuracy (0.47) were the strongest task-based predictors. In the combined model, classification accuracy reached 91.1%, primarily driven by PROMIS Depression (0.66) and Motivational Deficit (0.27). These findings suggest that app-based neuropsychological tasks may be useful as an adjunctive tool for early screening of subclinical depression and anxiety.

A closer inspection of the working-memory task indicates that abandonment tendency showed significant positive correlations with multiple self-report scales, including the PHQ-9 and GAD-7, and the between-group difference was also significant. Thus, higher depressive and anxiety symptoms were associated with a greater propensity to give up when task difficulty increased. This pattern is consistent with the mood–behavior model (45), which proposes that negative affect biases perceived task difficulty upward, leading to disengagement from effort mobilization. Physiological studies also suggest progressive disengagement as task demands rise, as the depression group—compared with the control group—showed significant decreases in cardiac engagement with increasing task difficulty (46, 80). Accordingly, abandonment tendency captured by the present task may serve as a behavioral index that sensitively detects subclinical disengagement from complex tasks. Given the clear correlations with multiple self-report measures and the significant group difference, this indicator appears to be clinically useful for early screening of subclinical depression and anxiety.

The auditory working memory index was positively correlated with self-esteem, and, in group comparisons, the subclinical group performed worse than the control group. Although not statistically significant, both auditory and visual working memory indices consistently showed negative associations with depression and anxiety scales. This trend conceptually aligns with prior studies suggesting that emotional difficulties can impair working memory efficiency (42, 43) and implies that our working memory task can capture some executive function decrements associated with depression and anxiety. Compared with the Corsi block-tapping task (81), on which the present task was based, our implementation is more demanding because it requires simultaneous processing of visual and auditory stimuli (i.e., a multitasking format). Whether the lack of statistically significant correlations for both modalities reflects the subclinical sample’s relatively mild symptom severity (as opposed to clinical groups) or the higher task difficulty of our implementation warrants further investigation in larger samples, including controls, subclinical participants, and patients diagnosed with MDD and generalized anxiety disorder.

Unexpectedly, reasoning accuracy on the risk-taking decision-making task showed a significant positive correlation with the PHQ-9, and the subclinical group outperformed the control group. This finding could potentially be attributed to task-specific strategies rather than a general cognitive advantage; for instance, the relatively higher accuracy in the subclinical group may reflect loss aversion. Specifically, stronger negative affect toward monetary loss may have increased exploration across cups and enhanced the motivation to discover the rule. Prior studies have also linked depression with heightened loss aversion (82). When individuals recognize that repeating the same choice under uncertainty may lead to loss, greater exploration can occur (83). However, given the unexpected nature of this result, these findings should be interpreted with caution as a possible manifestation of specific exploratory decision-making strategies under uncertainty. Further replication studies are required to confirm whether this pattern consistently appears in subclinical populations.

The motivational deficit index showed significant positive correlations with depression and anxiety scales, and the group difference was significant: Higher depression and anxiety were associated with slower button-pressing speed to obtain initial funds. This aligns with previous observations that diminished motivation and reward sensitivity in depression can impair cognitive task performance (84). As our measure of motivational deficitis derived from “button-press speed,” it likely reflects not only reward sensitivity but also processing speed. Accordingly, the group difference in motivational deficit may, in part, reflect the psychomotor retardation commonly observed in depression—a feature that reliably differentiates patients from controls (85, 86).

Decision variability in the risk-taking task showed significant positive correlations with depression and anxiety scales. Although not statistically significant, the subclinical group tended to exhibit higher variability than the control group. Greater depression and anxiety were associated with difficulty maintaining a consistent strategy and frequent switching between high- and low-risk options in response to negative feedback. This pattern is compatible with reinforcement sensitivity theory (53) and findings by Huang et al. (57) that individuals with a high degree of anxiety proneness are hypersensitive to punishment cues and prone to switching strategies.

By contrast, risk-taking tendency was neither significantly correlated with depression and anxiety nor directionally consistent, and the group difference was not significant. In our task, participants chose between options with gains/losses of 1,000 or 5,000 KRW and received percentile-based feedback after each choice. As the money was not actually paid out and percentile feedback only conveyed rank information (i.e., one’s standing), participants may have been less engaged, which could have masked the risk-averse tendencies often observed in depression and anxiety (87).

In the mood-induction word memory task, the short-term memory index showed negative directional associations with self-reported depression and anxiety (see **Supplementary Table 1**), and the mean score of the subclinical group was slightly lower than that of the control group; however, neither analysis reached statistical significance. These findings diverge from prior reports of short-term memory deficits in patients with depression (59). As reviewed by Marazziti et al. (59), short-term memory impairment in depression may be minimal and secondary to other dysfunctions (e.g., reduced motivation and cognitive initiative). Hammar and Årdal (88) reported mixed results regarding immediate verbal memory deficits in depression. As our sample comprised subclinical rather than clinical participants, the small performance disadvantages relative to the controls may not have achieved statistical significance.

Although not statistically significant and therefore requiring caution in interpretation, negative cognitive bias tended to show positive directional associations with depression scales (see **Supplementary Table 1**), and the subclinical group exhibited a slightly greater bias than the control group. This is consistent with prior evidence that depression is associated with attentional and interpretive biases toward negative information (89, 90). Given that mood-congruent recall may be less prominent in subclinical than in clinical depression (91), the weak associations and group differences observed here are not unexpected. As negative bias tends to increase with symptom severity (91, 92), the characteristics of our subclinical sample may have attenuated the effects. Within ethical bounds, stronger negative and positive mood inductions and careful calibration of the emotional intensity of word stimuli may improve sensitivity to mild negative bias in subclinical groups.

For the motor effort index, correlations with depression and anxiety were unexpectedly positive in direction (see **Supplementary Table 1**), and the subclinical group tended to pull the slingshot more strongly than the control group, although the results were not significant. Considering the overall task battery, the subclinical group showed a greater tendency to give up on the comparatively difficult working memory task, whereas they appeared to engage more vigorously during the relatively easy slingshot component that indexed motor effort. Subclinical participants may exert more effort on easy tasks and less effort—and even quit—on difficult tasks. This is consistent with the mood–behavior model (45), according to which individuals select effort levels based on perceived task difficulty; indeed, individuals with depression show higher cardiovascular reactivity than controls when tasks are easy, but lower reactivity when tasks are difficult (46, 80). Accordingly, to adequately capture the reduced energy in individuals with depression within smartphone app-based environments, tasks should incorporate procedures that quantify motor output at an intermediate level of difficulty—neither too easy nor too difficult. Adjusting task format or difficulty is likely to improve sensitivity to reduced energy (anergia); for example, using accelerometer sensors in smartphones or smartwatches, or employing virtual reality paradigms that require the execution of specified motor actions.

For the visual tracking ability index, associations with self-report scales were not directionally consistent (see **Supplementary Table 1**), and no group differences were observed, which runs counter to reports that people with depression perform worse on tasks analogous to the Trail Making Test Type A (TMT-A) (93). This null finding is highly informative for future task refinement. A key difference likely lies in the outcome definition. In our task, visual tracking ability was defined as the number of correct predictions of the ball’s goal after tracking its descent; by contrast, classical TMT-A performance relies primarily on completion time (66). It should be noted, however, that this interpretation remains a speculative conjecture by the researchers, as our study did not directly compare different metric formats. Future research is required to test the hypothesis that temporal measures, such as completion time, are more sensitive than binary outcomes for detecting subtle cognitive decrements in subclinical populations.

The response time indices yielded mixed results. Most response time measures were not significantly correlated with depression; the directions for some were positive and others negative (see **Supplementary Table 1**). Although processing-speed decrements are often associated with depression (24), the complexity of the present tasks (e.g., inference, threat cues, mood induction) may have made it more challenging to isolate “pure” processing speed, compared with paradigms such as the TMT (94) or the Wechsler Coding and Symbol Search subtests, which index processing speed more effectively and commonly reveal slowing in depression (95). Notably, in the working and word memory tasks, several (mostly not significant) response time indices tended to correlate negatively with anxiety, suggesting faster responding at higher anxiety levels. As faster responding may increase error rates (speed–accuracy trade-off) (96), these indices should be interpreted together with accuracy and may reflect impulsive decision tendencies under anxiety. Furthermore, interface-related motor demands might have introduced non-cognitive noise; for instance, in the working memory task, users had to re-click a previously selected hole to cancel a choice if interference occurred during the raccoon sequence, potentially confounding the measured reaction time. The heterogeneity of our subclinical group may have also contributed to these inconsistencies. Although not statistically significant, the diverging directional correlations observed between certain RT indices and depression versus anxiety scales suggest that grouping these distinct symptoms into a single entity might have obscured symptom-specific cognitive patterns.

Discriminant analysis was used to evaluate the feasibility of app-based neuropsychological tasks as a screening aid for subclinical depression and anxiety. With the four task variables that showed significant group differences, the overall classification accuracy reached 70.5% (67.0% with leave-one-out cross-validation), indicating an improvement of at least 17 percentage points over the 50% baseline. Relatively few studies have assessed the screening performance of neuropsychological tasks for depression or anxiety. For example, Baussay et al. (97) reported that the TMT has comparatively good discriminative ability for generalized anxiety disorder, although it is inferior to a self-report scale (GAD-7). Troyan and Levada (98) used word memory tasks and the TMT-B to classify MDD; however, discriminative performance varied widely by age. Given the well-known limitations of standalone self-report scales (e.g., recall bias), the incremental value of objective tasks is evident. In the present study, when neuropsychological tasks were combined with self-report measures, the classification accuracy for distinguishing controls from subclinical participants increased to 91.1%, with a sensitivity of 89.1% and a specificity of 93.0%. While these results are promising, they should be interpreted cautiously as preliminary and hypothesis-generating, given the exploratory nature of this study and the modest sample size. Nevertheless, these findings suggest the potential utility of using app-based neuropsychological tasks alongside self-report scales to screen for high risk of depression and anxiety, potentially compensating for the limitations of self-report measures alone.

### 4.1 Limitations and future directions

This study has several limitations. Regarding sample characteristics, first, because the subclinical classification relied on self-report measures (PHQ-9, GAD-7), generalization to clinical populations is limited. Second, we analyzed a combined subclinical group rather than separating depression and anxiety subgroups, which, alongside the relatively modest sample size, constrains disorder-specific inferences. Additionally, the potential influence of digital literacy and demographic variables such as age and gender on task performance cannot be entirely ruled out.

In terms of methodology and analysis, the cross-sectional design precludes causal inference regarding the observed associations. Furthermore, we did not apply formal corrections for multiple comparisons due to the exploratory nature of this preliminary study aimed at hypothesis generation; thus, results should be interpreted with caution. Finally, all tasks were administered in a fixed order to ensure procedural consistency, though this may have introduced order or fatigue effects. Future studies with larger, longitudinal samples and randomized task orders are needed to validate these preliminary findings.

As neuropsychological task performance can be affected by depression and anxiety severity (42, 67), the differences between the subclinical groups and the control group may be subtle. Accordingly, careful adjustment of task difficulty may be required to detect subclinical features. These results provide a valuable roadmap for refining task procedures and optimizing the battery by prioritizing discriminative variables, thereby enhancing screening efficiency and minimizing participant fatigue. Distinguishing between depression-only and anxiety-only subgroups will help identify task variables that are more specific to each condition and improve screening accuracy for subclinical states.

### 4.2 Conclusion

This study provides the first evidence of the validity of the developed smartphone app-based neuropsychological tasks and their feasibility as an adjunctive tool for early screening of subclinical depression and anxiety. Although these tasks appear promising as a supplemental screener for high-risk groups, accurate subclinical screening may require further adjustment for task difficulty and procedures, given the relatively modest neurocognitive impairment typical of subclinical populations. Additionally, a multimodal screening system that integrates self-report scales with biosignal indices collected during task performance (e.g., heart rate, voice, temperature, electrodermal activity), coupled with an appropriate classification algorithm, may further enhance performance.

## Data Availability

The raw data supporting the conclusions of this article will be made available by the authors, without undue reservation.
