# Supplementary material for: Feasibility of smartphone app-based neuropsychological tasks for screening people with subclinical depression and anxiety: a preliminary validation study
Source: Front Psychiatry. 2026 Mar 16;17:1773101. doi: 10.3389/fpsyt.2026.1773101 (PMC13033642; doi:10.3389/fpsyt.2026.1773101)
Supplement: Supplementary file 1 [file Table1.docx]

Supplementary Material

**Table S1. Correlations Among Study Variables**

| **Item** | **PHQ-9** | **PROMIS Dep** | **RSES** | **RRS** | **GAD-7** | **PROMIS Anx** | **ASI-3** | **DII** |
| --- | --- | --- | --- | --- | --- | --- | --- | --- |
| **1. V-WM** | -.01 | -.09 | .07 | .03 | .05 | -.03 | .12 | .02 |
| **2. A-WM** | -.17 | -.14 | **.20^*^** | -.11 | -.12 | -.15 | -.04 | -.08 |
| **3. AT** | **.25^**^** | .18 | **-.21^*^** | .17 | **.29^**^** | **.25^**^** | **.21^*^** | **.26^**^** |
| **4. WK-RT** | .10 | .10 | -.12 | .01 | -.07 | .03 | -.13 | .05 |
| **5. RA** | .**19^*^** | .17 | -.13 | .18 | .14 | .15 | .16 | .13 |
| **6. DV** | .**22^*^** | .17 | **-.19^*^** | .14 | **.32^**^** | **.23^*^** | .15 | .10 |
| **7. RTT** | .02 | .05 | .04 | .03 | .06 | -.05 | .05 | .08 |
| **8. MD** | .**20^*^** | **.22^*^** | -.14 | .06 | .10 | **.21^*^** | -.01 | .03 |
| **9. R-RT** | -.16 | -.15 | .13 | -.18 | -.17 | -.12 | **-.19^*^** | -.18 |
| **10. STM** | -.14 | -.11 | .09 | .01 | -.14 | -.12 | -.11 | .02 |
| **11. NCB** | .10 | .09 | -.07 | .12 | .00 | .07 | -.01 | .13 |
| **12. WD-RT** | -.10 | -.03 | .10 | **-.19^*^** | **-.21^*^** | -.10 | **-.21^*^** | -.16 |
| **13. ME** | .13 | .10 | -.09 | .17 | .14 | .18 | .16 | .16 |
| **14. VTA** | .01 | -.16 | .13 | -.04 | .07 | -.12 | -.04 | -.05 |
| **15. S-RT** | .06 | .10 | .01 | .01 | -.03 | .04 | -.08 | -.09 |

Note. *N* = 112. *p* < .05(^*^), *p* < .01(^**^). V-WM = Visual Working Memory; A-WM = Auditory Working Memory; AT = Abandonment Tendency; WK-RT = Working Memory Task Response Time; RA = Reasoning Accuracy; DV = Decision Variability (inverse indicator of Decision Consistency); RTT = Risk-Taking Tendency; MI = Motivational Deficit; R-RT = Risk-Taking Decision-Making Task Response Time; STM = Short-Term Memory; NCB = Negative Cognitive Bias; WD-RT = Word Memory Task Response Time; ME = Motor Effort; VTA = Visual Tracking Ability; S-RT = Soccer Task Response Time; PHQ-9 = Patient Health Questionnaire-9; PROMIS Dep = PROMIS Depression Short Form; RSES = Rosenberg Self-Esteem Scale; RRS = Ruminative Response Scale; GAD-7 = Generalized Anxiety Disorder-7; PROMIS Anx = PROMIS Anxiety Short Form; ASI-3 = Anxiety Sensitivity Index-3; DII = Distress Intolerance Index

Table S2. Detailed Psychometric Properties of the App-Based Task Variables.

| **Variable** | **Sub-indicator / Phase** | **Reliability** | **Method** |
| --- | --- | --- | --- |
| **Working Memory Task** |  |  |  |
| Visual Working Memory | Total (14 trials) | .58 | Split-half ($r_{sb})$ |
| Auditory Working Memory | Total (22 trials) | .80 | Split-half ($r_{sb})$ |
| Abandonment Tendency | Total (28 stages) | .72 | Cronbach’s α |
| **Risk-Taking Decision-Making Task** |  |  |  |
| Reasoning Accuracy | Total (20 trials) | .18 | Cronbach’s α |
|  | First half  (Trials 1-10) | -.19 | Cronbach’s α |
|  | Second half (Trials 11-20) | .22 | Cronbach’s α |
| Decision Variability | - | - | N/A (Aggregate) |
| Risk-Taking Tendency | - | - | N/A (Aggregate) |
| **Word Memory Task** |  |  |  |
| Short-Term Memory | Total (90 items) | .87 | Cronbach’s α |
|  | Neutral Words (30 items) | .77 | Cronbach’s α |
|  | Positive Words  (30 items) | .68 | Cronbach’s α |
|  | Negative Words  (30 items) | .74 | Cronbach’s α |
| Negative Cognitive Bias | Total (6 recall sessions) | .35 | Split-half ($r_{sb})$ |
| **Visual Tracking Task** |  |  |  |
| Visual Tracking Ability | Total (13 trials) | .78 | Split-half ($r_{sb})$ |
| Motor Effort | Total (13 trials) | .95 | Cronbach’s α |

Note.$r_{sb}$ = Spearman-Brown corrected split-half reliability. Reliability for Risk-Taking Tendency and Decision Variability was not calculated due to aggregate data storage. The negative α in the first half of Reasoning Accuracy reflects the exploratory trial-and-error phase, whereas the increase in the second half (.22) indicates stabilized performance after learning. Low NCB reliability (.35) is characteristic of proportion-based metrics, while its components (Positive/Negative recall) remain stable.
